# Supplementary material for: Curcumin Nanodiscs Improve Solubility and Serve as Radiological Protectants against Ionizing Radiation Exposures in a Cell-Cycle Dependent Manner
Source: Nanomaterials (Basel). 2022 Oct 15;12(20):3619. doi: 10.3390/nano12203619 (PMC9609432; doi:10.3390/nano12203619)
Supplement: Supplementary file 1 [file nanomaterials-12-03619-s001.zip › nanomaterials-1832832-supplementary.pdf]

Supplemental Figures

Supplemental Figure S1: Curcumin treatment increases Phospho-H3 positive fractions in MRC-5 cells.

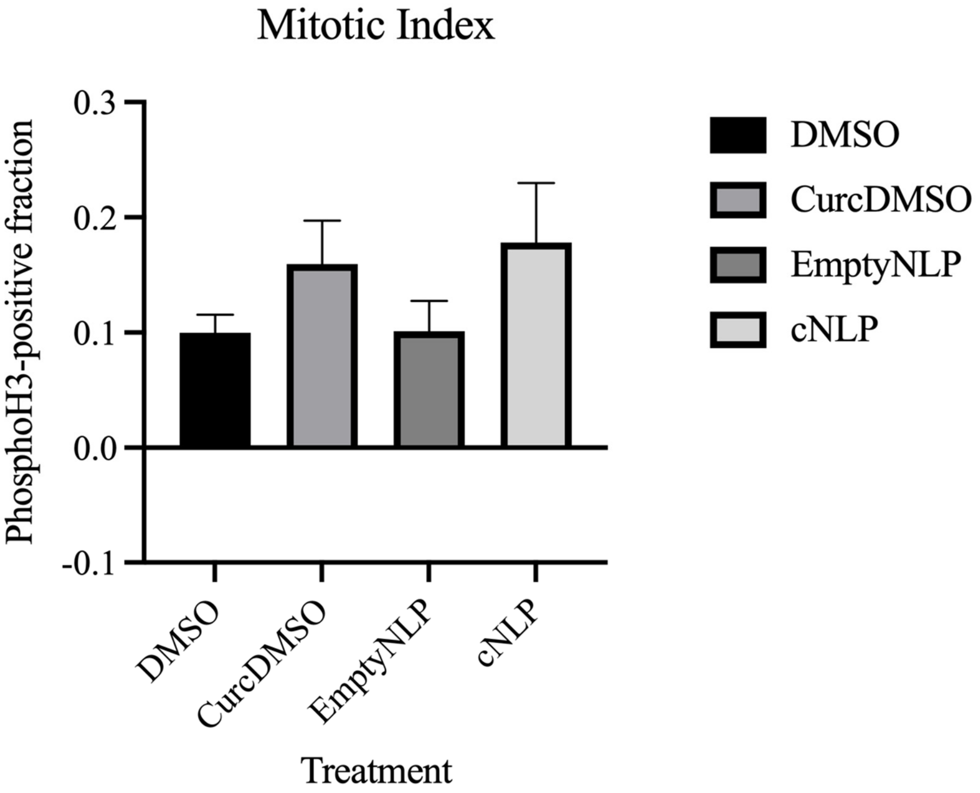

Supplemental Figure S2: Quantification of foci from AG05965/MRC-5 fibroblasts following IR exposure. Cells were pre-treated for 18 hours with cNLP (**A**), curDMSO (**B**), or 0.1% DMSO (**C**) prior to IR (50 cGy or SHAM). Cells were fixed 15 minutes, 2 hours, 6 hours, or 24 hours after irradiation and immunostained with DSB markers gamma-H2AX/pSer139 and 53BP1. All outliers have been removed. The black line represents the mean foci/cell per treatment group. Statistics are via one-way ANOVA.

A.

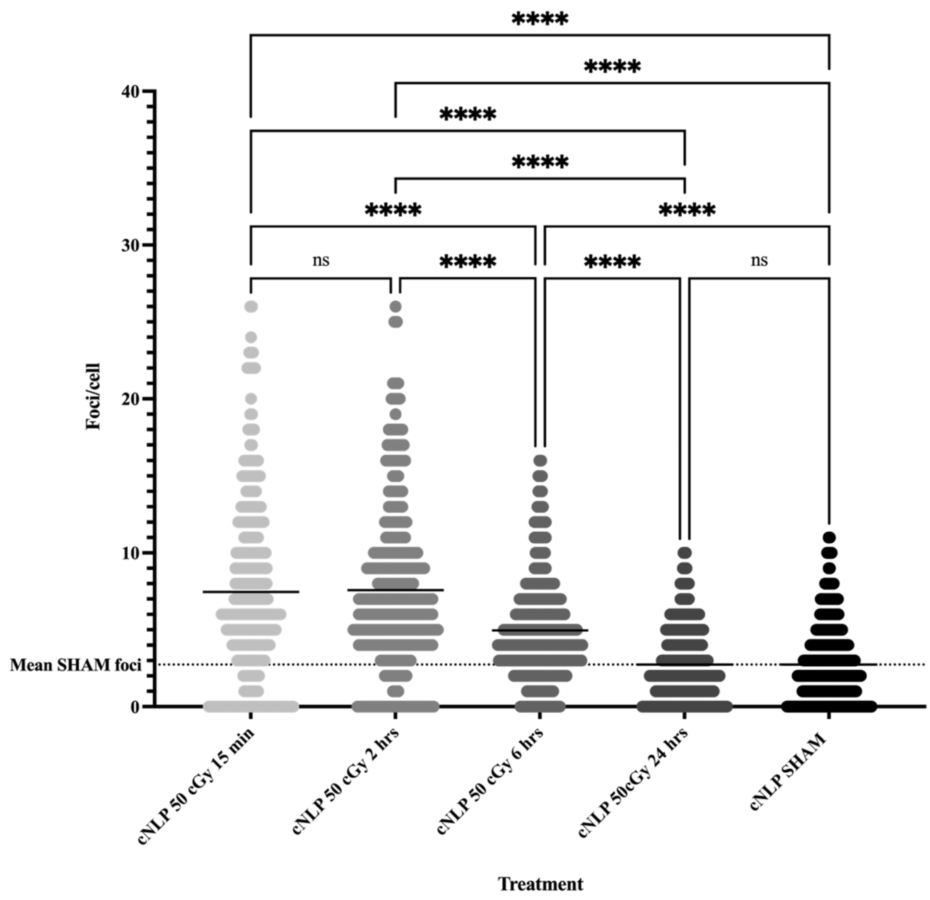

B.

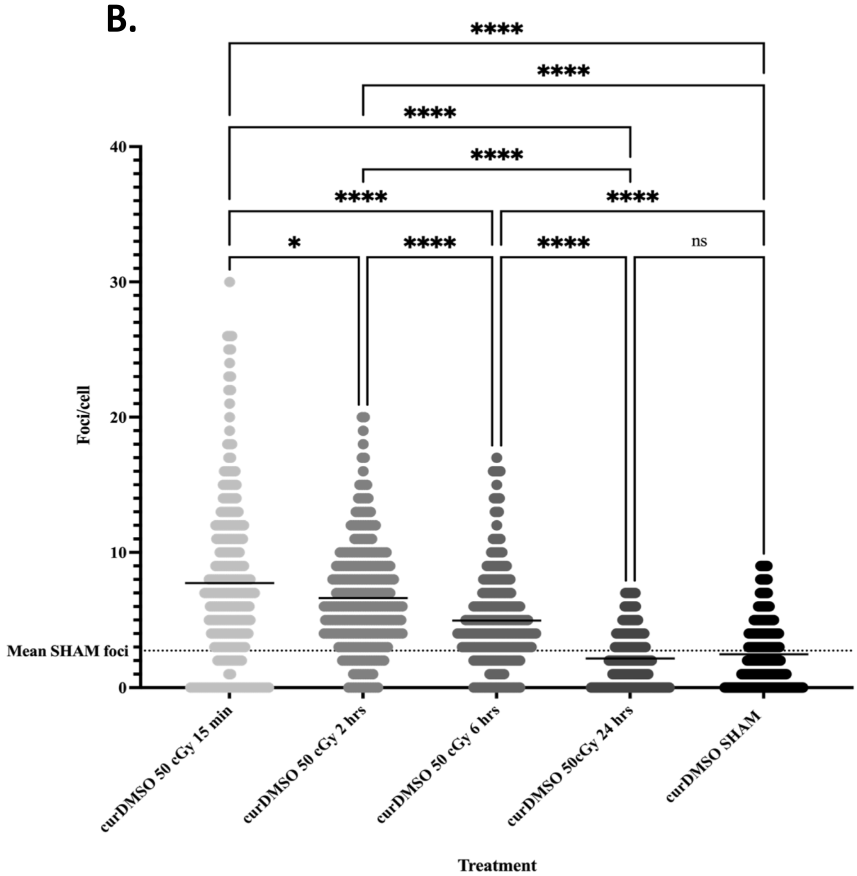

****
****
****
****
****
****
*
****
****
ns
40
30
20
10
0
Foci/cell
Mean SHAM foci
curDMSO 50 cGy 15 min
curDMSO 50 cGy 2 hrs
curDMSO 50 cGy 6 hrs
curDMSO 50cGy 24 hrs
curDMSO SHAM
Treatment

C.

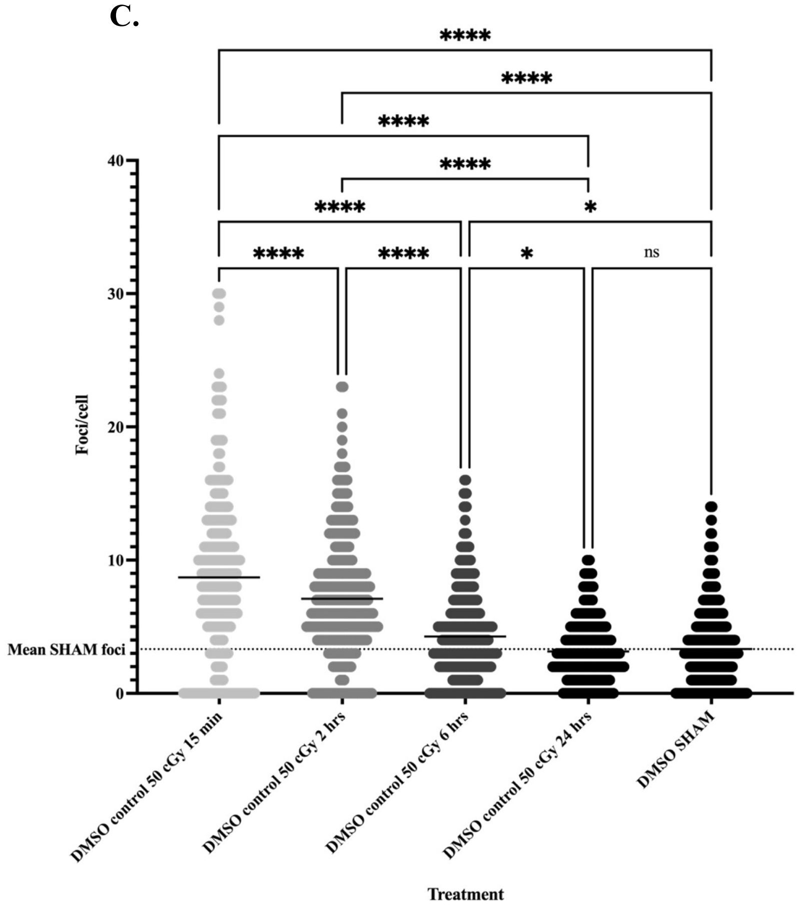

****
****
****
****
****
*
****
****
*
ns
40
30
20
10
0
Foci/cell
Mean SHAM foci
DMSO control 50 cGy 15 min
DMSO control 50 cGy 2 hrs
DMSO control 50 cGy 6 hrs
DMSO control 50 cGy 24 hrs
DMSO SHAM
Treatment
